# Supplementary material for: Sequential Injection Analysis for Automation and Evaluation of Drug Liberation Profiles: Clotrimazole Liberation Monitoring
Source: Molecules. 2021 Sep 12;26(18):5538. doi: 10.3390/molecules26185538 (PMC8467677; doi:10.3390/molecules26185538)
Supplement: Supplementary file 1 [file molecules-26-05538-s001.zip › molecules-1371598-supplementary.pdf]

molecules

Supplementary material

Sequential Injection Analysis for Automation and Evaluation of Drug Liberation Profiles: Clotrimazole Liberation Monitoring

Hana Sklenářová [1,*], Marek Beran [1], Lucie Novosvětská [1], Daniela Šmejkalová [2] and Petr Solich [1]

[1] Department of Analytical Chemistry, Faculty of Pharmacy in Hradec Králové, Charles University, 500 05 Hradec Králové, Czech Republic; beranma1@faf.cuni.cz (M.B.); lucie.novosvetska@gmail.com (L.N.); solich@faf.cuni.cz (P.S.)
[2] Contipro a.s., 561 02 Dolní Dobrouč, Czech Republic; daniela.smejkalova@contipro.com
* Correspondence: sklenarova@faf.cuni.cz; Tel.: +420-495-067-453

Table S1. Composition of the tested clotrimazole formulations.

**Clotrimazol AL 1% cream, 20 g**
1 g of cream contains 10 mg of clotrimazole Excipients: Sorbitan stearate, polysorbate 60, artificial whale, cetyl stearyl alcohol, benzyl alcohol, octyldodecanol, purified water
**Canesten cream** 1×20 g/200 mg
Clotrimazole 200 mg in 20 g cream. Excipients: sorbitan stearate, polysorbate 60, spermaceti, cetylstearyl alcohol, octyldodecanol, benzyl alcohol, purified water
**Delcore** – for 3.3% clotrimazole dispersion preparation
Lyophilizate with clotrimazole content corresponding to 3.3% while 5 mg is dissolved in 1 mL of phosphate buffer (0.1 mol L^{-1}, pH = 7.4)

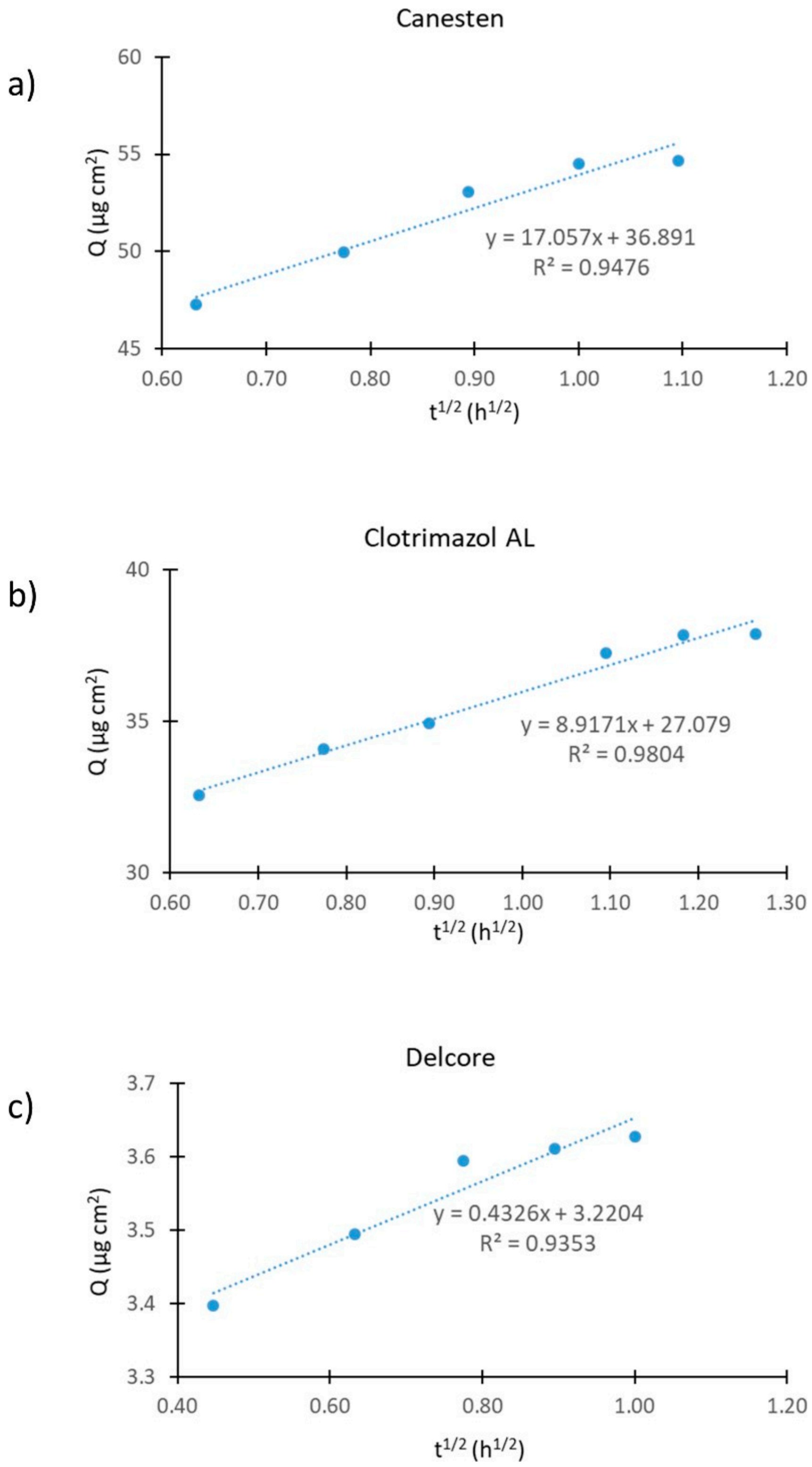

Figure S1. Linearization of liberated clotrimazole amount in three Franz cells experiment for. (**a**) Canesten, (**b**) Clotrimazol AL, and (**c**) Delcore formulations.

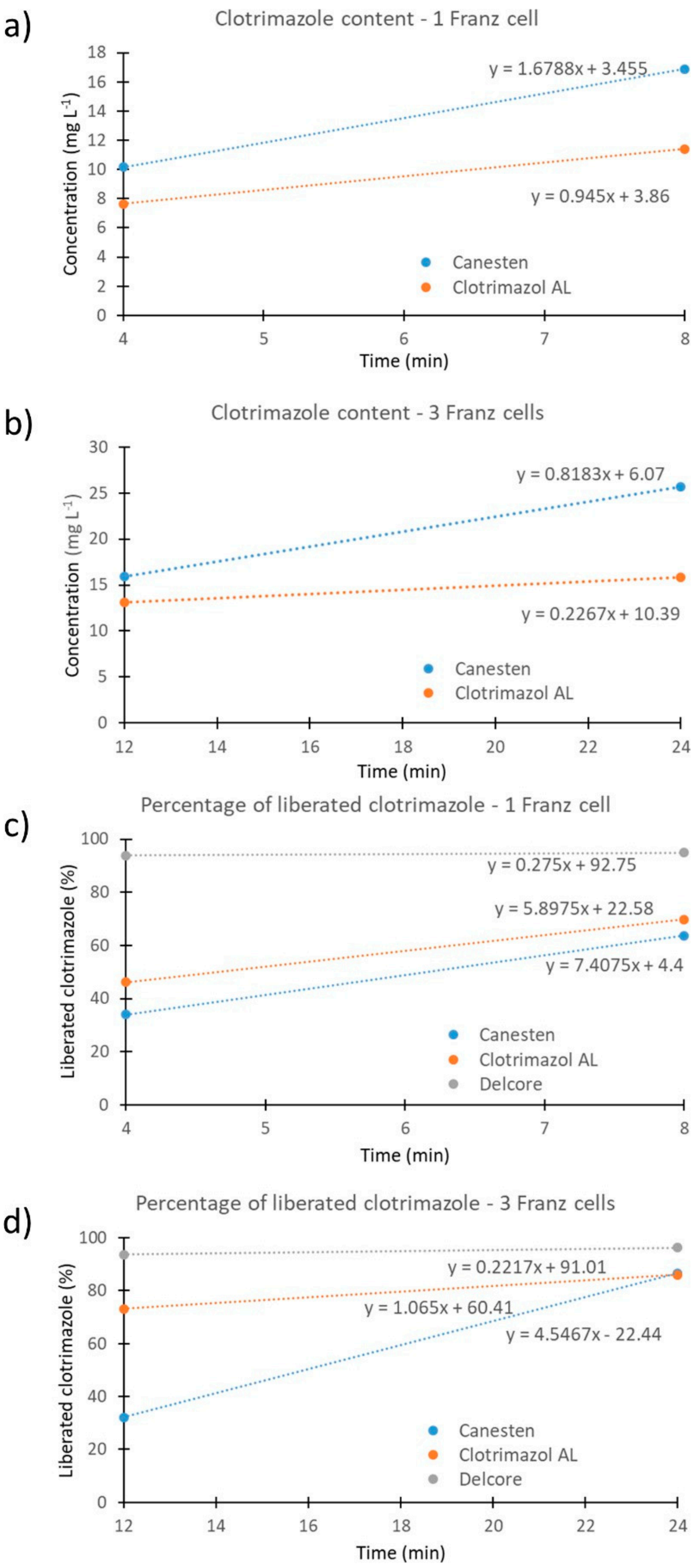

Figure S2. Evaluation based on initial rate of the second and third sampling points for clotrimazole content in (**a**) one Franz cell, (**b**) three Franz cells experiment, and percentage ofliberated clotrimazole in (**c**) one Franz cell, and (**d**) three Franz cells experiment.
